# Causal association of immune cells and endometriosis: a Mendelian randomization study

**DOI:** 10.3389/fendo.2024.1397670

**Published:** 2024-05-29

**Authors:** Xingyi Fang, Qinghua Deng, Haili Yang, Zhaohua Yan, Zhen Peng, Yuheng Zhao, Tantan Liao, Ziying Tu, Jia Liu, Li Liu, Lin Zou, Honghua He

**Affiliations:** ^1^ Reproductive Medical Center, Affiliated Hospital of Guangdong Medical University, Zhanjiang, Guangdong, China; ^2^ Department of Gynaecology, The Second Affiliated Hospital of Guangdong Medical University, Zhanjiang, Guangdong, China; ^3^ Department of Obstetrics and Gynecology, Affiliated Hospital of Guangdong Medical University, Zhanjiang, Guangdong, China; ^4^ Graduate School of Guangdong Medical University, Zhanjiang, Guangdong, China; ^5^ Department of Hematology, Affiliated Hospital of Guangdong Medical University, Zhanjiang, Guangdong, China

**Keywords:** immune cells, endometriosis, MR analysis, SNP, sensitivity analyses

## Abstract

**Objective:**

To investigate the causal effect of immune cells on endometriosis (EMS), we performed a Mendelian randomization analysis.

**Methods:**

Mendelian randomization (MR) uses genetic variants as instrumental variables to investigate the causal effects of exposures on outcomes in observational data. In this study, we conducted a thorough two-sample MR analysis to investigate the causal relationship between 731 immune cells and endometriosis. We used complementary Mendelian randomization (MR) methods, including weighted median estimator (WME) and inverse variance weighted (IVW), and performed sensitivity analyses to assess the robustness of our results.

**Results:**

Four immune phenotypes have been found to be significantly associated with the risk of developing EMS: B cell %lymphocyte (WME: OR: 1.074, p = 0.027 and IVW: OR: 1.058, p = 0.008), CD14 on Mo MDSC (WME: OR: 1.056, p =0.021 and IVW: OR: 1.047, p = 0.021), CD14+ CD16− monocyte %monocyte (WME: OR: 0.947, p = 0.024 and IVW: OR: 0.958, p = 0.011), CD25 on unsw mem (WME: OR: 1.055, p = 0.030 and IVW: OR: 1.048, p = 0.003). Sensitivity analyses confirmed the main findings, demonstrating consistency across analyses.

**Conclusions:**

Our MR analysis provides compelling evidence for a direct causal link between immune cells and EMS, thereby advancing our understanding of the disease. It also provides new avenues and opportunities for the development of immunomodulatory therapeutic strategies in the future.

## 1 Introduction

Endometriosis (EMS) is a chronic inflammatory condition characterized by the presence of endometrium-like tissue outside the uterus (1). Patients with endometriosis have 25–40% concurrent infertility, while 40–87% experience chronic pelvic pain (2). Genetic predisposition (3), hormonal irregularities (4), environmental influences (5), immune dysregulation (6) and unique anatomical configurations (7) are recognized as potential risk factors associated with the onset of endometriosis. The available evidence suggests that immune dysfunction plays an important role in both the pathogenesis of endometriosis and the manifestation of its clinical symptoms (8).

Disturbances in immune homeostasis can create a favorable environment for implantation, proliferation and angiogenesis of ectopic endometrial tissue (9). The abnormal activation of immune cells such as B cells (10), T cells (11), natural killer (NK) cells (12), Dendritic cells (DC) (13), Monocytes (14) and macrophages (15) leads to increased levels of various inflammatory factors, autoantibodies and cytokines. However, findings on the relationship between immune cells and EMS have been inconsistent.

A systematic review that synthesized the results of 22 selected trials found that the majority of trials reported an increased number and/or activation of B cells in endometriosis. However, seven trials did not find a significant difference, while two trials showed a reduced number of B cells (16). These discrepancies may be due to a limited sample size, to flaws in the design of the studies, and to confounding factors that are beyond the scope of the existing studies.

While quantitative changes in CD4+ T cells have been investigated in numerous studies, there have been conflicting results regarding the frequency of CD4+ T cells in both peripheral blood (PB) and/or peritoneal fluid (PF) when comparing individuals with endometriosis (EM) to healthy women (17).

NK cells have been proposed to have a significant influence on the pathogenesis of the disease, potentially either promoting tolerance or inhibiting the survival, implantation and proliferation of endometrial cells (18). Several studies have reported a significant reduction in the levels of CD56+ NK cells in peritoneal fluid samples from women diagnosed with endometriosis (19, 20). However, alternative research perspectives posit that there are no discernible distinctions in CD56+ NK cell levels within the peritoneal fluid between patients and control groups (21, 22).

The growth and vascularization of endometriosis is dependent on the presence of endogenous dendritic cells, which infiltrate endometriotic lesions and promote endothelial cell migration through the secretion of proangiogenic factors (23). Hey-Cunningham et al. identified variations in dendritic cell populations both locally in endometrial tissue and systemically in the circulation in women with endometriosis, with stage-specific associations within the endometrium (24). However, the impact of dendritic cells on the development of endometriosis lesions in mouse models has been inconsistent (25–27).

Monocytes, macrophages and dendritic cells (DCs) act as antigen-presenting cells (APCs), stimulate T cells and secrete a variety of inflammatory mediators that can modulate immune responses (28).The phagocytic function of peripheral monocytes is reduced in patients with endometriosis compared to healthy women, which may contribute to the observed immunological changes in the disease, as well as being influenced by the presence of ectopic endometrial lesions (29).

Macrophages are classified according to their activation pathway as either ‘classically activated’ (M1) or ‘alternatively activated’ (M2) macrophages. While it is widely accepted that M2 macrophages promote the progression of endometriosis, with M1 macrophages playing a lesser role (30, 31), Takebayashi et al. (32) and Vallvé-Juanico et al. (33) present a contrasting perspective, suggesting that M1 macrophages are the predominant macrophage population in the endometrium of patients with endometriosis. The temporal distribution of macrophages in ectopic endometrial tissue remains uncertain. Braun et al. (34) found a decrease in macrophage numbers only during the early proliferative phase in patients with endometriosis, whereas Khan et al. (35) found higher macrophage numbers in patients with endometriosis than in non-endometriosis patients during all phases. In addition, Berbic et al. (36) reported an increase in macrophage numbers throughout the proliferative phase in patients with endometriosis.

**Figure 1:**
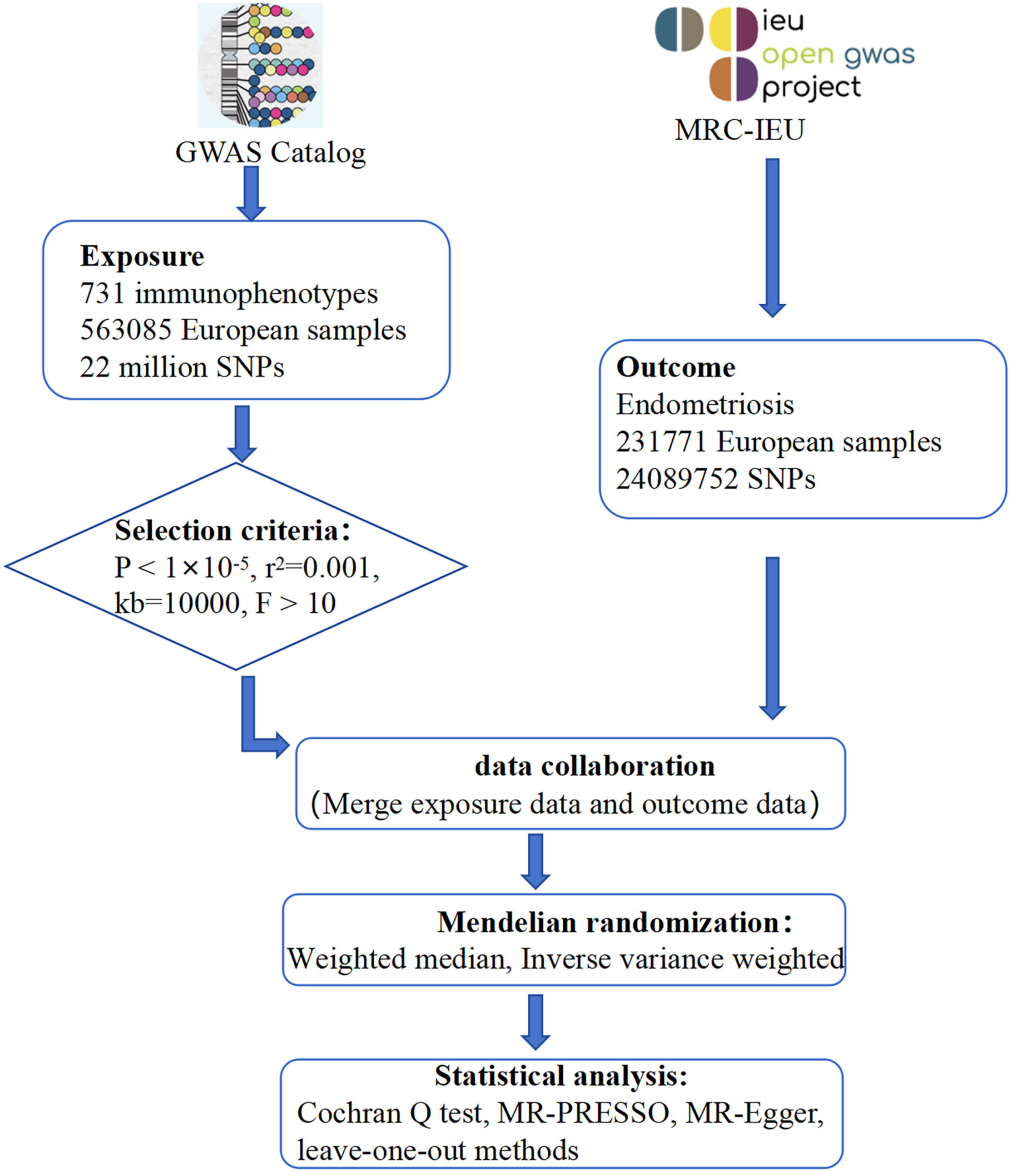
Overall design of the study.

Mendelian randomization (MR) is a well-established epidemiological method that uses genetic studies to elucidate causality (37). MR uses single nucleotide polymorphisms (SNPs) identified through genome-wide association studies (GWAS) as instrumental variables, effectively simulating randomized controlled trials (RCTs) and mitigating biases associated with confounding and reverse causation (38). Previous observational studies have suggested numerous associations between immune cell characteristics and endometriosis. In this study, we further elucidate the causal relationship between immune cell characteristics and endometriosis from a genetic perspective using MR analysis, thereby providing more targeted strategies for future interventions and treatments. This has significant implications for the prevention, diagnosis and treatment of endometriosis (**Figure 1**).

## 2 Materials and methods

### 2.1 Study design

In this study, we first assessed 731 immune cell types as potential exposure factors, using single nucleotide polymorphisms (SNPs) significantly correlated with these cells as instrumental variables. Endometriosis was then considered as the outcome variable in our analysis. The analysis was performed using a two-sample Mendelian randomization (MR) approach. To ensure the reliability of the results, we tested for heterogeneity, pleiotropy and sensitivity analysis.

### 2.2 GWAS data sources

We downloaded data on 731 immune cells from the GWAS Catalogue website (https://www.ebi.ac.uk/gwas/home) with PubMed ID 32929287. This dataset has accession numbers from GCST0001391 to GCST0002121 and includes data from 563,085 European samples, covering approximately 22 million SNPs (39).

We obtained genetic data on endometriosis from the MRC-IEU OpenGWAS platform (https://gwas.mrcieu.ac.uk/). Specifically, we retrieved GWAS data on endometriosis patients with dataset ID ebi-a-GCST90018839. This dataset includes 4,511 cases and 227,260 controls from European samples. The data allowed the estimation of genetic associations between 24089752 SNPs and endometriosis.

### 2.3 Selection of instrumental variables

SNPs serve as instrumental variables (IVs) in this Mendelian randomization (MR) analysis aimed at assessing the causal relationship between exposure and outcome (40). The selection of IVs in this study must meet three key assumptions: (1) IVs have a strong correlation with exposure (e.g. immune cell characteristics); (2) IVs are not directly associated with outcome (e.g. endometriosis); (3) IVs are independent of confounding variables.

Following current research standards, and allowing for the possibility of missing some immune cells due to overly high thresholds, SNPs significantly associated with immune cells (P < 1×10^-5) were selected from the GWAS summary data for further analysis (41–44). Parameters such as r^2 = 0.001 and kb=10000 were set to eliminate the influence of linkage disequilibrium in the analysis. To reduce bias and eliminate weak instrumental variables, only SNPs with an F-statistic greater than 10 were retained for subsequent analysis. SNPs potentially associated with confounding factors were identified and excluded using PhenoScanner V2 (http://www.phenoscanner.medschl.cam.ac.uk/).

### 2.4 Statistical analysis

The Inverse Variance Weighted (IVW) method is widely regarded as the standard approach for performing MR analysis. The IVW method assumes that all instrumental variables are valid. Its principle is to aggregate the Wald ratio estimates for each instrumental variable to estimate causality. If heterogeneity is detected, a random effects model is used, otherwise a fixed effects model is used (45). The weighted median method (WME) requires that more than 50% of the instrumental variables correspond to true SNPs. Compared to other methods, the weighted median method requires a smaller sample size and guarantees less bias and a lower type I error rate (46).

To estimate causal effects in MR analysis, this study used both the WME and IVW methods. If both methods give results of P < 0.05, this is considered to indicate a direct causal relationship, thus ensuring a high level of confidence in the results. All analyses were performed using the TwoSampleMR package within R version 4.3.1.

### 2.5 Sensitivity analysis

Sensitivity analysis is of paramount importance in Mendelian randomization studies to identify and address potential pleiotropy. In this study, sensitivity analyses were performed using the Cochran Q test, MR-PRESSO, MR-Egger and leave-one-out methods. The presence of heterogeneity in the instrumental variables was assessed using the Cochran Q test and the MR-PRESSO global heterogeneity test, with P > 0.05 indicating no heterogeneity. The multi-effect test was performed using the MR-Egger regression method, and the intercept term P<0.05 indicates horizontal pleiotropy. The leave-one-out method involved systematically removing each SNP in turn and then recalculating the results using the remaining SNPs. This procedure was used to assess whether the effect of individual SNPs disproportionately influenced the association.

### 2.6 Reverse MR analysis

Research on endometriosis as an Exposure Factor and the immune cells as the Outcome. According to the screening criteria (p = 5e-08, r2 = 0.001, kb = 10000), SNPs significantly associated with endometriosis were selected. The reverse causal relationship between endometriosis and immune cells was then analyzed using the IVW, MR-Egger, WME, WM and Simple Mode methods.

## 3 Results

### 3.1 Exploration of the causal effect of immunophenotypes on EMS

**Figure 2:**
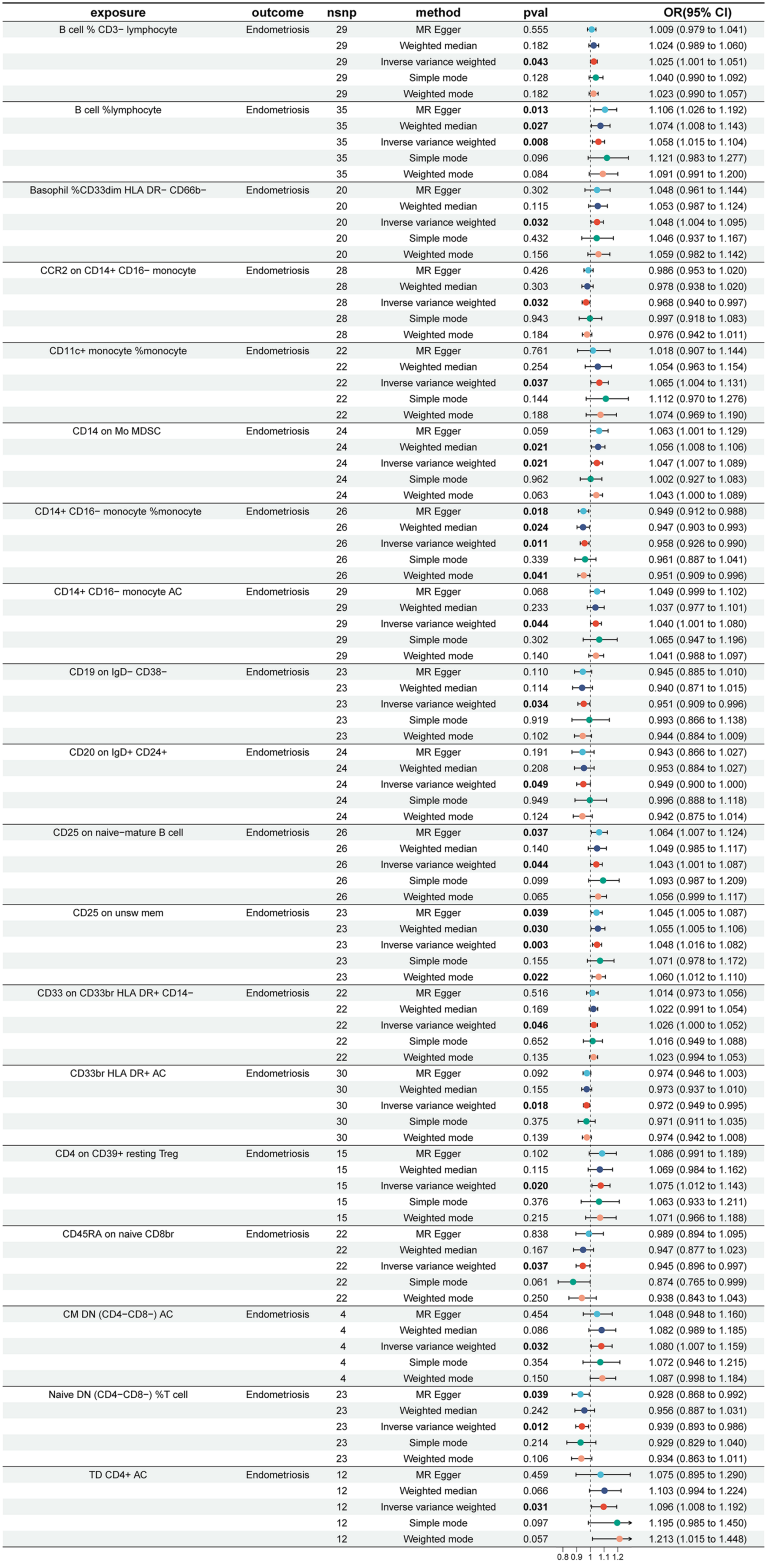
Impact of 19 Immune Cells on EMS (IVW: p < 0.05). nsnp, number of single nucleotide polymorphisms; OR, odds ratio; CI, confidence interval.

**Figure 3:**
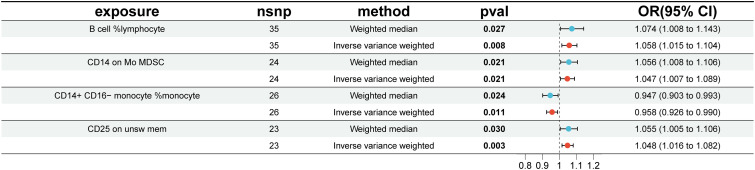
Impact of 4 Immune Cells on EMS (WME and IVW: p < 0.05). nsnp, number of single nucleotide polymorphisms; OR, odds ratio; CI, confidence interval.

To investigate the causal effects of 731 immune cells on endometriosis, a two-sample MR analysis was performed. The IVW method identified a total of 19 immune cells associated with the onset of endometriosis (**Figure 2**). After further screening, only four immune cells remained that met the criteria of P < 0.05 in both the WME and IVW methods (**Figure 3**).

**Figure 4:**
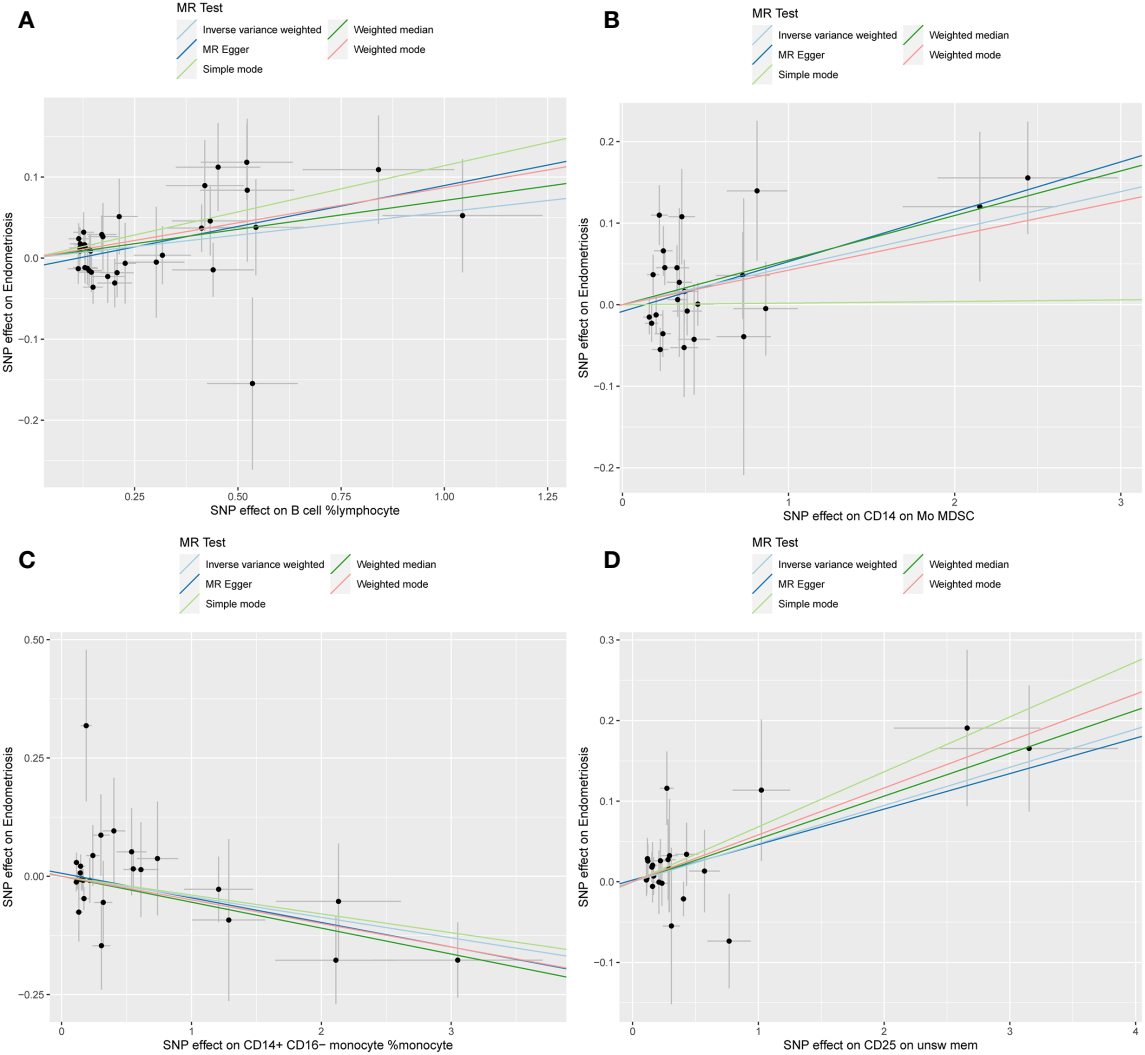
Scatter plot illustrating the relationship between the SNP effect size of causal immune traits (x-axis) and the corresponding effect size estimates of EMSs (y-axis). **(A)** B cell %lymphocyte, **(B)** CD14 on Mo MDSC, **(C)** CD14+ CD16− monocyte %monocyte, **(D)** CD25 on unsw mem.

The WME and IVW MR analyses identified four immunological features significantly associated with the presence of endometriosis: B cell %lymphocyte (WME: OR: 1.074, p = 0.027 and IVW: OR: 1.058, p = 0.008), CD14 on Mo MDSC (WME: OR: 1.056, p =0.021 and IVW: OR: 1.047, p = 0.021), CD14+ CD16− monocyte %monocyte (WME: OR: 0.947, p = 0.024 and IVW: OR: 0.958, p = 0.011), CD25 on unsw mem (WME: OR: 1.055, p = 0.030 and IVW: OR: 1.048, p = 0.003). As shown in the scatter plot, the characteristics (e.g. B cell %lymphocyte, CD14 on Mo MDSC and CD25 on unsw mem) were positively associated with EMS(**Figures 4A, B, D**), whereas the characteristics (i.e. CD14+ CD16- monocyte %monocyte) was negatively associated with EMS (**Figures 4C**).

### 3.2 Sensitivity analysis

**Figure 5:**
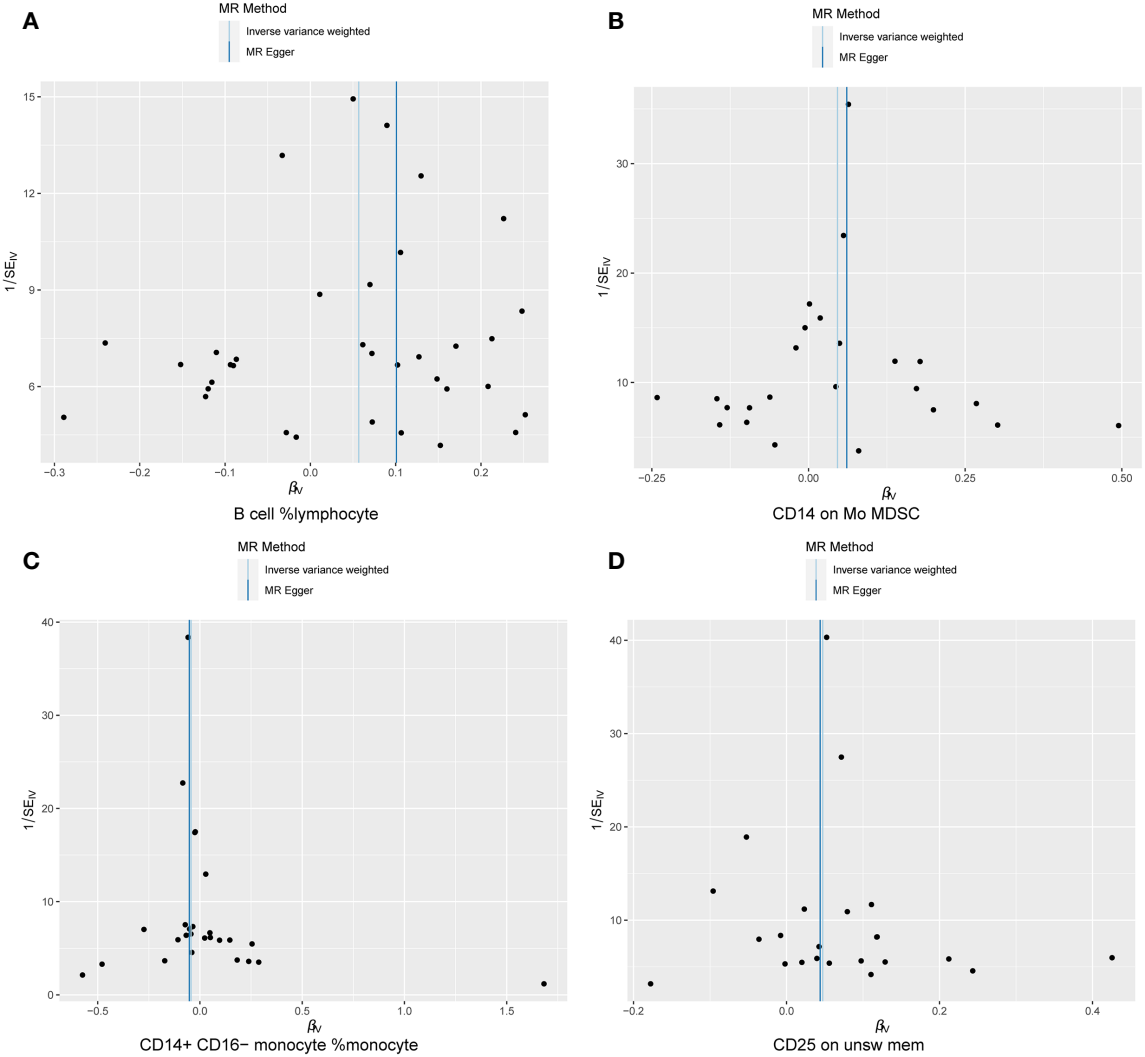
Sensitivity results Funnel plot. **(A)** B cell %lymphocyte, **(B)** CD14 on Mo MDSC, **(C)** CD14+ CD16− monocyte %monocyte, **(D)** CD25 on unsw mem.

**Figure 6:**
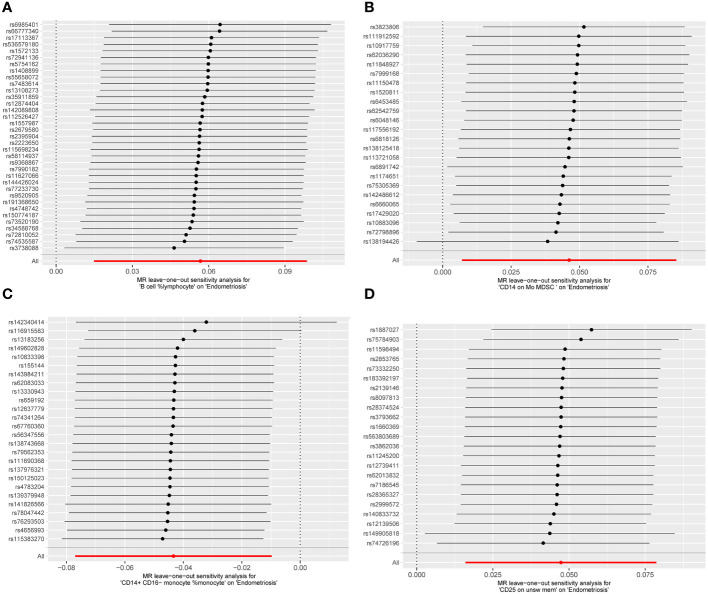
MR leave−one−out sensitivity analysis. **(A)** B cell %lymphocyte, **(B)** CD14 on Mo MDSC, **(C)** CD14+ CD16− monocyte %monocyte, **(D)** CD25 on unsw mem.

Cochran Q test results indicated no heterogeneity between SNPs (**Supplementary Table 1**). The result of the MR-Egger intercept test was that horizontal pleiotropy had no effect on the MR analysis results (**Supplementary Table 2**). The funnel plot shows a symmetrical distribution on both sides. This indicates that there is no bias in the results of the MR analysis (**Figure 5**). There were no outlier SNPs in the MR-PRESSO global test results (**Supplementary Table 3**). Sensitivity analysis using the leave-one-out method showed that the results of the MR analysis were not influenced by individual SNPs (**Figure 6**).

### 3.3 Causal Effects of EMS on the four immune cells

**Figure 7:**
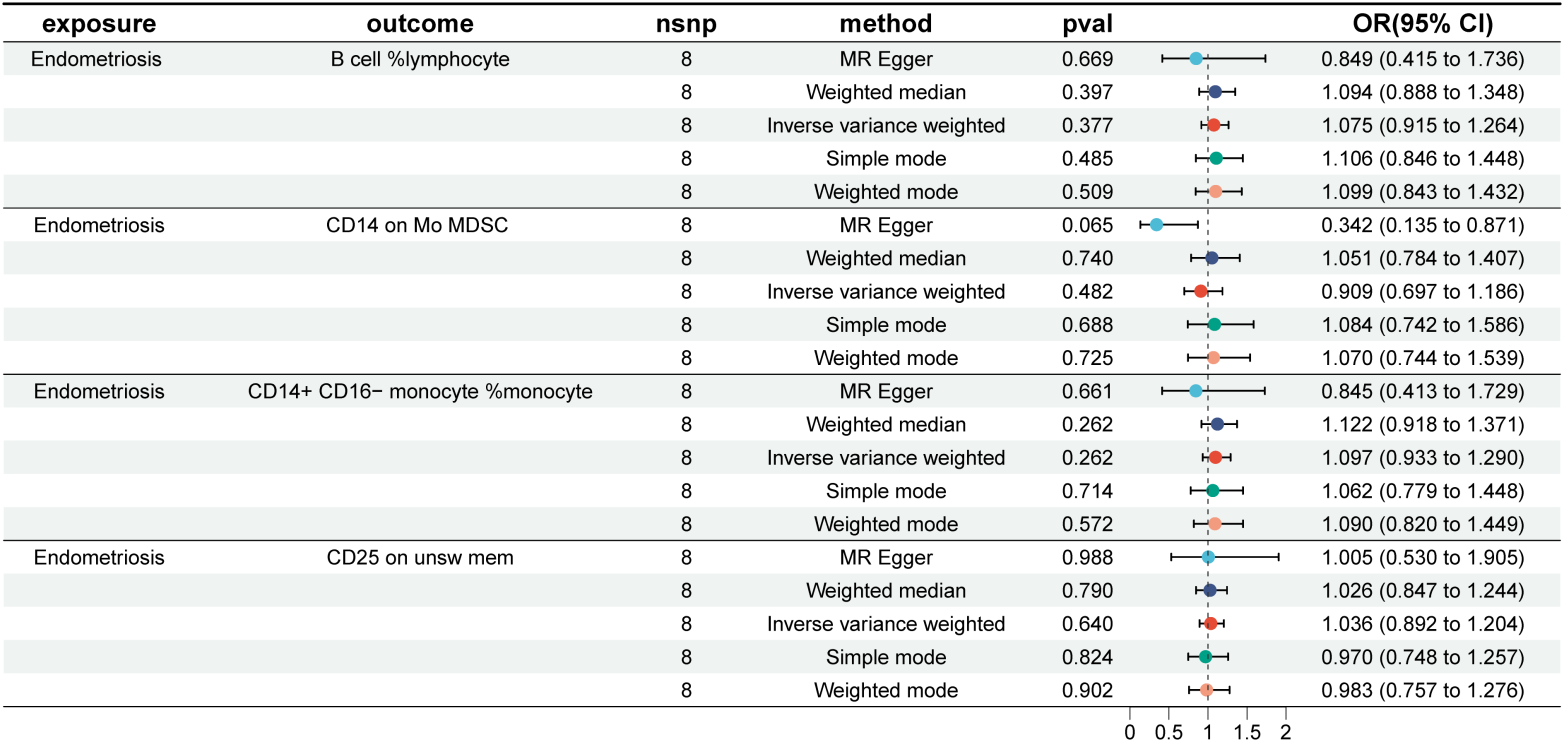
EMS and the four immune cells. nsnp, number of single nucleotide polymorphisms; OR, odds ratio; CI, confidence interval.

In the reverse causal analysis, none of the five MR analysis methods suggested a significant causal relationship between EMS and the four immune cells (P>0.05) (**Figure 7**). The remaining 15 immune cells identified by the IVW method showed only one with a reverse causal relationship with endometriosis: CD11c+ monocyte %monocyte (IVW: OR: 1.271, p = 0.004) (**Supplementary PDF1**).

## 4 Discussion

We investigated the causal relationships between 731 immune cell traits and endometriosis using large, publicly available genetic datasets. In this study, the IVW method identified 19 immune cells, while the combined WME method identified four immune phenotypes that were significantly associated with EMS causality: ‘B cell %lymphocyte’, ‘CD14 on Mo MDSC’, ‘CD14+ CD16- monocyte %monocyte’, and ‘CD25 on unsw mem’. However, there is no reverse causal relationship between endometriosis and these four immune cells.

Our results suggest a significant correlation between two types of B cells, ‘B cell %lymphocyte’ and ‘CD25 on unsw mem’, and an increased risk of endometriosis.

‘B cell %lymphocyte’ is the proportion of B cells in the total number of lymphocytes. Lymphocytic immune cells are essential for endometrial cells to survive and proliferate (47). A systematic review shows that the majority of studies have documented an increase in both the number and activity of B cells in the peripheral blood, endometrial tissue or peritoneal fluid of people with endometriosis (16). In addition, Andrew J. Shih et al. performed single-cell RNA sequencing (scRNA-Seq) analysis comparing endometrial tissue obtained from freshly collected menstrual fluid (MF) samples from 33 subjects. They found a significant increase in B cells in the shed endometrium of individuals diagnosed with endometriosis (p = 5.8 × 10–^6^) (48). Studies by Nothnick et al. have shown elevated serum levels of autoantibodies of varying specificity, including anti-endometrial and antisperm antibodies, in women diagnosed with endometriosis (49). The dysfunctional behavior of B lymphocytes in endometriosis is characterized by an increase in the production and quantity of antibodies, particularly autoantibodies, which can lead to immune evasion by endometrial cells, thereby accelerating disease progression (50). Antsiferova et al. used peripheral blood or uterine endometrial lymphocytes from healthy women as controls and observed a significant increase in the amount of pan-B cells, especially B lymphocytes of the B-1 subset, and the level of activated B lymphocytes in the ectopic endometrium (50). B-1 cells, derived from fetal B lymphocytes, have unique developmental and functional characteristics. They demonstrate the ability to generate natural, polyreactive antibodies that are critical for maintaining tissue homeostasis and enhancing immune defense (51). The increased production of autoantibodies resulting from B-1 cell activation may serve as a mechanism to facilitate evasion of immune surveillance by endometrial cells. A key feature of B-1 cells is their synthesis of low-affinity poly-reactive immunoglobulins, which have the ability to recognize a wide range of autoantigens and show cross-reactivity with many bacterial antigens, including polysaccharides and lipopolysaccharides (52). Lebovic et al. suggested that anti-endometrial autoantibodies may mask the antigenic determinants of endometrial cells, potentially shielding them from immune cell attack (53).

‘CD25 on unsw mem’ means that CD25 is expressed on non-switched memory B cells. Switched memory B cells originate from the germinal center and consist of isotype-switched IgG, IgA, IgE and pre-switched IgM+ only cells. Conversely, non-switched memory B cells are antigen-experienced B cells expressing either IgM+IgD+ or the smaller subset expressing only IgD+ (IgM-) (54). B cells expressing CD25 spontaneously secrete immunoglobulins of the IgA, IgG and IgM subclasses and have an enhanced migratory capacity compared to CD25(-) B cells (55). The increased migratory capacity of endometrial stromal cells (ESCs) is a fundamental determinant in the development of functional endometrium-like tissue outside the uterine cavity in EMS (56).

In an experimental rat model of endometriosis, Dogan et al. observed a significant reduction in the volume of endometriotic implants following treatment with Rituximab, a B-cell antibody (57). Although several studies have demonstrated aberrant production of endometrial autoantibodies in endometriosis, there is no consensus about the concentration of B cells (in eutopic and ectopic endometrium, circulating blood and/or peritoneal fluid) and their roles in this disorder (58). Future studies of ‘B cell %lymphocytes’ and ‘CD25 on unsw mem’ may provide additional insights into the pathogenesis of endometriosis and facilitate consensus in this field.

Our results also showed a positive correlation between increased levels of ‘CD14 on Mo MDSC’ and increased risk of EMS, while an increase in ‘CD14+ CD16- monocyte %monocyte’ was inversely associated with EMS risk.

Zhang et al. found that the number of myeloid-derived suppressor cells (MDSCs) decreases in human patients after laparoscopic surgery, while depletion of MDSCs in mouse models significantly reduces endometriotic lesions and adoptive transfer of MDSCs restores lesion growth. This suggests a proactive recruitment process of MDSCs during endometriosis, which may promote lesion survival and progression (59). Researchers identify the lack of definitive markers for human MDSCs as a major hurdle, contributing to delays in characterizing and conducting *in situ* studies of this complex immunosuppressive population (60). The ‘CD14 on Mo MDSC’ subset of MDSCs may serve as a focal point for future research efforts, facilitating a deeper understanding of the pathogenic mechanisms underlying endometriosis.

‘CD14+ CD16- monocyte %monocyte’ indicates the proportion of CD14+ CD16- monocytes in the total monocyte population. In healthy people, about 90% of monocytes are characterized as being CD14 positive and CD16 negative, known as CD14+CD16- classical monocytes (61). In inflammatory contexts, classical monocytes migrate into tissues where they differentiate into either macrophages or dendritic cells (62). In this capacity, they perform various functions, including the removal of apoptotic bodies, the promotion of angiogenesis and the restoration of tissue integrity, thereby contributing to the reduction of lesions (63). Research by Hogg et al. shows that endometriosis triggers a sustained recruitment of monocytes into the peritoneal cavity and an increased influx of monocytes into the large peritoneal macrophage (LpM) reservoir. In this context, monocyte-derived LpMs have been observed to exert a protective influence against the progression of endometriosis lesions (64). Although ‘CD14+ CD16- monocyte %monocyte’ has rarely been reported in endometriosis, our study results provide new insights into the differentiation of monocytes into macrophages and the activation of monocytes in endometriosis, thus providing valuable guidance for future research efforts.

Our study used two-sample MR analysis, drawing on extensive GWAS datasets of approximately 231771 individuals, ensuring robust statistical power. The study’s conclusions relied on genetic instrumental variables with a predefined threshold of P<0.05 for both WME and IVW MR analysis methods, aiming to enhance result robustness against potential issues like horizontal pleiotropy and other confounding factors. The identification of four immune cell types (‘B cell %lymphocyte’, ‘CD14 on Mo MDSC’, ‘CD14+ CD16- monocyte %monocyte’, and ‘CD25 on unsw mem’) elucidated the interaction patterns between the immune system and endometriosis and provided additional valuable data on the immune environment surrounding the complex pathogenic molecular mechanisms of endometriosis. Our findings suggest the potential for integrating checkpoint inhibitors with strategies targeting B cells and MDSC in future immunotherapy studies for endometriosis. However, this study also has several limitations. First, the two-sample MR analysis was based on summary data from the GWAS, which lacked detailed demographic information and clinical characteristics of the participants, precluding subsequent subgroup analyses. As a result, we were unable to explore potential variations in the causal relationship between immune cells and endometriosis across different phases of the menstrual cycle. Secondly, the predominantly European origin of the study sample limits the generalizability of the findings to other populations. Future research could include clinical trials in different countries to achieve more precise immunotherapy interventions.

## 5 Conclusions

In summary, our MR analysis provides robust evidence for a causal link between immune cells and susceptibility to EMS. This finding holds great promise for informing clinical decisions regarding disease prognosis and treatment modalities, while also paving the way for novel drug development efforts.

## Data availability statement

The datasets presented in this study can be found in online repositories. The names of the repository/repositories and accession number(s) can be found in the article/**Supplementary Material**.

## Author contributions

XF: Conceptualization, Validation, Writing – original draft. QD: Conceptualization, Writing – original draft. HY: Conceptualization, Writing – original draft. ZY: Data curation, Writing – original draft. ZP: Formal Analysis, Writing – original draft. YZ: Investigation, Writing – original draft. TL: Methodology, Writing – original draft. ZT: Project administration, Writing – original draft. JL: Software, Writing – original draft. LL: Software, Writing – review & editing. HH: Resources, Writing – review & editing. LZ: Funding acquisition, Writing – review & editing.
