# Supplementary material for: Causal association of immune cells and endometriosis: a Mendelian randomization study
Source: Front Endocrinol (Lausanne). 2024 May 29;15:1397670. doi: 10.3389/fendo.2024.1397670 (PMC11167632; doi:10.3389/fendo.2024.1397670)
Supplement: Supplementary file 4 [file DataSheet_1.pdf]

Causal Effects of endometriosis on the 19 immune cells

exposure	outcome	nsnp	method	pval	OR(95% CI)
Endometriosis	B cell %CD3− lymphocyte	8	MR Egger	0.800	0.907 (0.438 to 1.875)
		8	Weighted median	0.228	1.130 (0.926 to 1.379)
		8	Inverse variance weighted	0.342	1.081 (0.920 to 1.270)
		8	Simple mode	0.344	1.163 (0.869 to 1.555)
		8	Weighted mode	0.350	1.151 (0.874 to 1.514)
Endometriosis	B cell %lymphocyte	8	MR Egger	0.669	0.849 (0.415 to 1.736)
		8	Weighted median	0.397	1.094 (0.888 to 1.348)
		8	Inverse variance weighted	0.377	1.075 (0.915 to 1.264)
		8	Simple mode	0.485	1.106 (0.846 to 1.448)
		8	Weighted mode	0.509	1.099 (0.843 to 1.432)
Endometriosis	Basophil %CD33dim HLA DR− CD66b−	8	MR Egger	0.994	1.003 (0.426 to 2.361)
		8	Weighted median	0.784	1.035 (0.808 to 1.326)
		8	Inverse variance weighted	0.824	1.023 (0.837 to 1.251)
		8	Simple mode	0.970	1.007 (0.695 to 1.461)
		8	Weighted mode	0.853	1.034 (0.733 to 1.459)
Endometriosis	CCR2 on CD14+ CD16− monocyte	8	MR Egger	0.631	0.835 (0.415 to 1.681)
		8	Weighted median	0.849	1.020 (0.833 to 1.249)
		8	Inverse variance weighted	0.878	0.988 (0.847 to 1.153)
		8	Simple mode	0.588	1.087 (0.815 to 1.450)
		8	Weighted mode	0.609	1.079 (0.816 to 1.426)
Endometriosis	CD11c+ monocyte %monocyte	8	MR Egger	0.919	1.038 (0.521 to 2.067)
		8	Weighted median	**0.014**	1.290 (1.054 to 1.580)
		8	Inverse variance weighted	**0.004**	1.271 (1.082 to 1.494)
		8	Simple mode	0.122	1.295 (0.971 to 1.727)
		8	Weighted mode	0.128	1.287 (0.966 to 1.715)
Endometriosis	CD14 on Mo MDSC	8	MR Egger	0.065	0.342 (0.135 to 0.871)
		8	Weighted median	0.740	1.051 (0.784 to 1.407)
		8	Inverse variance weighted	0.482	0.909 (0.697 to 1.186)
		8	Simple mode	0.688	1.084 (0.742 to 1.586)
		8	Weighted mode	0.725	1.070 (0.744 to 1.539)
Endometriosis	CD14+ CD16− monocyte %monocyte	8	MR Egger	0.661	0.845 (0.413 to 1.729)
		8	Weighted median	0.262	1.122 (0.918 to 1.371)
		8	Inverse variance weighted	0.262	1.097 (0.933 to 1.290)
		8	Simple mode	0.714	1.062 (0.779 to 1.448)
		8	Weighted mode	0.572	1.090 (0.820 to 1.449)
Endometriosis	CD14+ CD16− monocyte Absolute Count	8	MR Egger	0.565	0.822 (0.437 to 1.545)
		8	Weighted median	0.324	1.103 (0.907 to 1.342)
		8	Inverse variance weighted	0.084	1.139 (0.983 to 1.321)
		8	Simple mode	0.706	1.057 (0.802 to 1.393)
		8	Weighted mode	0.736	1.051 (0.797 to 1.386)
Endometriosis	CD19 on IgD− CD38− B cell	8	MR Egger	0.306	1.647 (0.687 to 3.947)
		8	Weighted median	0.957	1.006 (0.803 to 1.261)
		8	Inverse variance weighted	0.870	1.018 (0.827 to 1.253)
		8	Simple mode	0.832	1.045 (0.708 to 1.542)
		8	Weighted mode	0.692	1.078 (0.756 to 1.536)
Endometriosis	CD20 on IgD+ CD24+ B cell	8	MR Egger	0.971	1.012 (0.533 to 1.924)
		8	Weighted median	0.336	0.906 (0.740 to 1.108)
		8	Inverse variance weighted	0.112	0.885 (0.762 to 1.029)
		8	Simple mode	0.477	0.901 (0.688 to 1.182)
		8	Weighted mode	0.476	0.907 (0.702 to 1.170)
Endometriosis	CD25 on naive−mature B cell	8	MR Egger	0.541	0.806 (0.419 to 1.549)
		8	Weighted median	0.567	1.059 (0.870 to 1.290)
		8	Inverse variance weighted	0.820	1.018 (0.874 to 1.186)
		8	Simple mode	0.641	0.928 (0.687 to 1.254)
		8	Weighted mode	0.551	1.105 (0.809 to 1.510)
Endometriosis	CD25 on unsw mem	8	MR Egger	0.988	1.005 (0.530 to 1.905)
		8	Weighted median	0.790	1.026 (0.847 to 1.244)
		8	Inverse variance weighted	0.640	1.036 (0.892 to 1.204)
		8	Simple mode	0.824	0.970 (0.748 to 1.257)
		8	Weighted mode	0.902	0.983 (0.757 to 1.276)
Endometriosis	CD33 on CD33+ HLA DR+ CD14−	8	MR Egger	0.764	1.221 (0.353 to 4.227)
		8	Weighted median	0.264	1.186 (0.879 to 1.599)
		8	Inverse variance weighted	0.286	1.159 (0.884 to 1.520)
		8	Simple mode	0.613	1.127 (0.724 to 1.755)
		8	Weighted mode	0.549	1.138 (0.760 to 1.704)
Endometriosis	CD33+ HLA DR+ Absolute Count	8	MR Egger	0.876	1.074 (0.457 to 2.521)
		8	Weighted median	0.878	1.021 (0.782 to 1.333)
		8	Inverse variance weighted	0.868	0.983 (0.804 to 1.202)
		8	Simple mode	0.812	1.046 (0.731 to 1.498)
		8	Weighted mode	0.919	1.019 (0.719 to 1.443)
Endometriosis	CD4 on CD39+ resting CD4 regulatory T cell	8	MR Egger	0.346	1.452 (0.711 to 2.966)
		8	Weighted median	0.975	0.996 (0.800 to 1.241)
		8	Inverse variance weighted	0.750	1.028 (0.869 to 1.215)
		8	Simple mode	0.826	1.042 (0.733 to 1.481)
		8	Weighted mode	0.867	1.035 (0.704 to 1.520)
Endometriosis	CD45RA on naive CD8+ T cell	8	MR Egger	0.604	1.214 (0.606 to 2.435)
		8	Weighted median	0.258	0.889 (0.724 to 1.090)
		8	Inverse variance weighted	0.469	0.942 (0.800 to 1.108)
		8	Simple mode	0.430	0.876 (0.643 to 1.194)
		8	Weighted mode	0.436	0.880 (0.649 to 1.192)
Endometriosis	Central Memory CD4−CD8− T cell Absolute Count	8	MR Egger	0.470	0.803 (0.459 to 1.403)
		8	Weighted median	0.405	0.930 (0.784 to 1.103)
		8	Inverse variance weighted	0.537	0.960 (0.842 to 1.094)
		8	Simple mode	0.354	0.872 (0.665 to 1.143)
		8	Weighted mode	0.363	0.885 (0.692 to 1.132)
Endometriosis	Naive CD4−CD8− T cell %T cell	8	MR Egger	0.674	1.155 (0.609 to 2.190)
		8	Weighted median	0.997	1.000 (0.839 to 1.192)
		8	Inverse variance weighted	0.805	0.982 (0.852 to 1.132)
		8	Simple mode	0.889	1.020 (0.783 to 1.329)
		8	Weighted mode	0.652	1.062 (0.827 to 1.363)
Endometriosis	Terminally Differentiated CD4+ T cell Absolute Count	8	MR Egger	0.865	1.062 (0.543 to 2.077)
		8	Weighted median	0.477	0.931 (0.763 to 1.135)
		8	Inverse variance weighted	0.895	0.989 (0.846 to 1.158)
		8	Simple mode	0.544	0.899 (0.647 to 1.248)
		8	Weighted mode	0.536	0.899 (0.651 to 1.240)

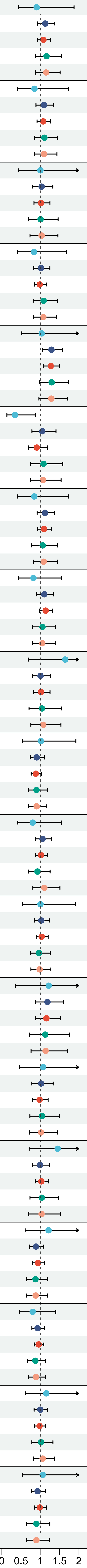

Note: P < 0.05 indicates a correlation between endometriosis and the production of this immune cell.
